# Coral Reef Fish Rapidly Learn to Identify Multiple Unknown Predators upon Recruitment to the Reef

**DOI:** 10.1371/journal.pone.0015764

**Published:** 2011-01-10

**Authors:** Matthew D. Mitchell, Mark I. McCormick, Maud C. O. Ferrari, Douglas P. Chivers

**Affiliations:** 1 Australia Research Council Centre of Excellence for Coral Reef Studies and School of Marine and Tropical Biology, James Cook University, Townsville, Australia; 2 Department of Environmental Science and Policy, University of California Davis, Davis, Davis, California, United States of America; 3 Department of Biology, University of Saskatchewan, Saskatoon, Canada; University of Western Ontario, Canada

## Abstract

Organisms often undergo shifts in habitats as their requirements change with ontogeny.

Upon entering a new environment, it is vitally important to be able to rapidly assess predation risk. Predation pressure should selectively promote mechanisms that enable the rapid identification of novel predators. Here we tested the ability of a juvenile marine fish to simultaneously learn the identity of multiple previously unknown predators. Individuals were conditioned with a ‘cocktail’ of novel odours (from two predators and two non-predators) paired with either a conspecific alarm cue or a saltwater control and then tested for recognition of the four odours individually and two novel odours (one predator and one non-predator) the following day. Individuals conditioned with the ‘cocktail’ and alarm cue responded to the individual ‘cocktail’ odours with an antipredator response compared to controls. These results demonstrate that individuals acquire recognition of novel odours and that the responses were not due to innate recognition of predators or due to a generalised response to novel odours. Upon entering an unfamiliar environment prey species are able to rapidly assess the risk of predation, enhancing their chances of survival, through the assessment of chemical stimuli.

## Introduction

Most organisms live under the constant threat of predation throughout their lives [1]. Antipredator behaviours are energetically expensive and reduce time available for other important activities such as foraging, mating and resource defence [1], [2]. As such, prey must minimise the risk of predation whilst maximising their energetic input to promote growth, reproductive success and ultimately, fitness [3], [4]. Prey must therefore be able to reliably identify potential predators, their associated risk and subsequently respond in a way that will optimise the balance between these conflicting demands.

The risks associated with predation vary with both time and space and will change throughout a prey's life [5]. Most organisms undergo ontogenetic shifts during their development, often resulting in individuals switching habitats in order to exploit superior food resources, shelter and establish or enhance mating opportunities [6]. On entering a new habitat, individuals are exposed to a new community of predators, some of which may be unknown or represent a different predation risk [7], [8]. The composition of potential predators within a given habitat will also change as fish grow and switch prey guilds [9], [10], or as environmental changes introduce new predators [11]. Predators themselves are also highly variable in space and time, ensuring that the risk of predation is in constant flux. Thus, prey should be dynamic and flexible in their antipredator behaviour.

Prey individuals must be able to develop antipredator strategies that can be adapted to match the current predation risk. The need to adapt antipredator strategies to their current environment explains why fixed innate antipredator strategies are uncommon amongst prey fish [12], [13]. Learning allows individuals to associate novel predators with danger and fine tune their antipredator responses to local environments, reducing the cost of unnecessary antipredator behaviours [1]. There is now extensive research showing that prey from a variety of taxa use learning to recognise predators and enhance their antipredator responses, including examples from fish, amphibians, reptiles, molluscs, mammals and birds [14], [15].

In aquatic environments, prey fish are able to access information about local predation risk from environmental cues using their visual, olfactory and mechanical senses [16]. Predator identity can be learnt through socially transmitted information [17], direct encounters with predators [18], [19], or indirectly, by associating a predator's odour with an alarm cue (chemical released by mechanical damage to the skin during a predation event) [20]. Chemosensory information provides reliable information about the identity and potential threat of unknown predators, as alarm cues are only released during direct predation encounters between predators and prey [21]. Fish are able to learn the identity of a novel predator by associative learning when the predator odour is presented simultaneously with an alarm cue released by mechanical damage to the skin of a conspecific [12], [22]. Indeed, the association between a novel odour and an alarm cue is so strong that after a single encounter, prey will respond with an antipredator response to the novel odour alone. This association can last several months [23]. Furthermore, these associations can even be made using alarm cues from heterospecific fishes in the same prey guild [12].

The majority of previous studies have investigated the ability of prey to acquire recognition of just one predator at a time under various conditions. However, few environments contain a single predator, with most prey exposed to several predators at any one time [24], [25]. Prey fish must be able to recognise any new potential risk of predation as fast and efficiently as possible. Learning multiple predators simultaneously would allow fast recognition of predators in a way that maximises time available for fitness promoting activities [26]. Darwish et al. [27] conditioned glowlight tetras, *Hemigrammus erythrozonus*, to a ‘cocktail’ of odours containing two predators and one non-predator subsequently demonstrating that tetras learnt to recognise each of the individual odours. They then proceeded to show that odours learnt in this way still confer a survival benefit. Currently it is unknown if these findings can be generalised across all fish or if they are specific to glowlight tetras.

Coral reefs are among the most biodiverse places on earth and provide a habitat for a rich assortment of fish including a vast array of predators, which can account for up to 50% of biomass in some reef communities [28]. After a pelagic developmental period, most juvenile reef fish return to coral reefs with little or no experience of the predators they will encounter. Mortality due to predation may reach nearly 60% during the first 2 days post settlement [29]. Recruiting juveniles must therefore learn the identity of local predators rapidly to survive. Recent studies have demonstrated that both juvenile and adult coral reef fish utilise chemical alarm cues to assess predation risk and to learn the identity of previously unknown predators through associative learning [30], [31]. We tested the lemon damselfish (*Pomacentrus moluccensis*) at the end of the pelagic larval phase to see if they could learn the identity of multiple predators during a single conditioning event. Naïve *P. moluccensis* were conditioned with a ‘cocktail’ of odours from two predators and two non-predators paired with a damage-released skin extract (alarm cue) from a conspecific or a seawater control. After conditioning, they were tested for recognition of each odour in the ‘cocktail’, as well as the odour of a novel predator and the odour of a novel non-predator.

## Methods

### Ethics Statement

This research was undertaken with approval of the James Cook University animal ethics committee (permit: A1067) and according to the University's animal ethics guidelines.

### Study species

Lemon damselfish, *Pomacentrus moluccensis*, are common planktivorous coral reef fish, found throughout the Indo-Pacific region and the Great Barrier Reef. They are particularly abundant on reefs around our study area, Lizard Island, Northern Great Barrier Reef, Australia (14°40′S, 145°28′E). Like many marine organisms, they undergo a planktonic phase, lasting 29 d, after which they settle to the reef [32]. At the time of settlement, they reach ∼10 mm in length and are preyed upon by multiple predators [33].

### Collection and maintenance

All fish were collected at Lizard Island during November and December 2009. *Pomacentrus moluccensis* recruits were collected from light traps (see small trap design, [34]) moored overnight near the reef crest, during the summer larval recruitment pulse. Recruits were captured prior to settling, 50–100 m away from the reef crest. The predators we used are associated with reefs, not open waters [35], and hence, the recruits should be naïve to the predators. *Pomacentrus moluccensis* were maintained in a 60 l aquarium (64.5×41.3×39.7 cm) supplied with aerated seawater and maintained at ambient seawater temperatures (29°C) under a 14∶10 light dark photoperiod. Fish were fed *ad libitum* twice a day with freshly hatched *Artemia* sp. and supplemented with 5/8 NRD marine food pellets (Spectrum Aquaculture). Fish were maintained in the aquaria for at least one day and a maximum of two weeks prior to being placed in experimental tanks.

Three known larval fish predators, brown dottyback, *Pseudochromis fuscus* (Family Pseudochromidae), clearfin lizardfish, *Synodus dermatogenys* (Synodontidae), batu wrasse, *Coris batuensis* (Labridae), and three non-fish predators, picasso triggerfish, *Rhinocanthus aculeatus* (Balistidae), sand goby, *Amblyeleotris steinitzi* (Gobiidae), and bluespot butterflyfish, *Chaetodon plebeius* (Chaetodontidae), were collected from the lagoon at Lizard Island using hand nets, barrier nets and anaesthetic clove oil mixed with alcohol and seawater. The fish were maintained as described above in 32 l aquaria (43.2×32.4×30.5 cm). Fish were fed twice a day with thawed bait squid.

### Stimulus preparation

Fresh alarm cues were prepared each day, 10 min prior to the conditioning phase. Six *P. moluccensis* were sacrificed by a quick blow to the head and placed in a plastic disposable Petri dish. Fifteen superficial vertical cuts were made along each side of the body of each fish with a scalpel blade. Each fish was then rinsed in 15 ml of seawater, yielding a total volume of 90 ml of alarm cues from the six fish. This solution was filtered through filter paper to remove any solid material prior to use.

Odours were prepared from pairs of *P. fuscus* (57 and 79 mm standard length (SL)), *S. dermatogenys* (93 and 102 mm SL), *C. batuensis* (124 and 86 mm SL), *R. aculeatus* (109 and 63 mm SL), *A. steinitzi* (65 and 53 mm SL) and *C. plebeius* (68 and 70 mm SL). Pairs of each species was placed in individual 32 l flow-through aquaria (43.2×32.4×30.5 cm). Fish were fed squid twice a day for two days and then starved for two days to remove any potential alarm cues present in their guts [36]. On the fourth day, each pair of fish from the same species was placed in a 32 l stimulus collection tank filled with 10 l of seawater, an airstone, and left undisturbed for 6 h. Following this period, the fish were moved back into the original holding tanks and the water from each stimulus collection tank was bagged in either 360 ml or 30 ml aliquots and frozen for later use.

### General experimental approach

Our experiment consisted of two phases: a conditioning phase followed by a testing phase. During the conditioning phase, we conditioned individual *P. moluccensis* to recognize a cocktail of four fish odours by exposing them to 120 ml of ‘cocktail’ odours (30 ml from each of the four ‘cocktail’ species; *P. fuscus*, *S. dermatogenys*, *R. aculeatus* and *C. plebeius*) paired with 15 ml of either conspecific alarm cues (true conditioning) or a water control (pseudo-conditioning). The next day, the fish were tested for their response to one of the four fish odours present in the cocktail or alternatively, the odour of two novel species (*C. batuensis*, *A. steinitzi*). We tested 15 fish in each of our 12 treatments (2 conditioning groups×6 odours tested). Although it would have been more rigorous to test for a response to saltwater as a control for the injection process, time constraints and animal limitations prevent us from doing so. In addition, several studies on Pomacentrid fish have demonstrated that they do not respond to the injection process [19], [30], [31]. If our larvae have the ability to learn to recognize individual predators from a cocktail mix, then we predict that they would display an antipredator response to each of the four species originally present in the cocktail, but would not respond to the odour of the two novel species. Recent studies have suggested that larval reef fish have an innate recognition of some predators [37], [38]. If that is the case, we predict that our larvae would respond more strongly to the two predatory species (*P. fuscus*, *S. dermatogenys*) than the two non-predatory species (*R. aculeatus*, *C. plebeius*). Additionally, we predict that they would also display an antipredator response when exposed to the ‘novel’ odour of a predator, *C. batuensis*, but not to the odour of a novel non-predator, *A. steinitzi*.

### Observation tanks

Conditioning and recognition trials were conducted in 13-l flow-through aquaria (36×21×20 cm, mean flow-though  = 0.6 litres/min). Each tank had a 3 cm layer of sand and a small terracotta pot (5 cm diameter) for shelter at one end and an air stone at the opposite end. Two injection tubes (a feeding tube and a stimulus tube) were attached to the airstone tube with their ends placed just above the stone to aid rapid dispersal of the chemical stimuli. The injection tubes allowed the food and stimuli to be introduced with minimal disturbance to the fish. A 4×6 grid was drawn onto the front of each tank. Each tank was surrounded on three sides with black plastic to visually isolate the fish and a black plastic curtain was hung in front of the tanks to create an observation blind.

### Conditioning phase

Single *P. moluccensis* were placed into each tank to acclimate overnight and then conditioned between 1000 h and 1130 h the following day. Prior to conditioning, odours from each of the four ‘cocktail’ species were thawed and mixed together to form the ‘cocktail’ of odours containing an equal amount of odour from each species. Prior to conditioning, the flow-through system was turned off to prevent the stimuli from flushing out. After a few minutes, we injected either 15 ml of alarm cue or 15 ml of seawater paired with 120 ml of cocktail odours. The fish were left undisturbed for 1 hr, after which the flow-through system was turned on again. We conditioned a total of 180 individuals, 12 fish per day.

### Recognition trials

Trials were conducted between 0730 h and 1430 h, the day after conditioning. Each trial consisted of an initial 5 min feeding period, a 5 min pre-stimulus observation and 5 min post-stimulus observation. Prior to the start of the trials, the flow-through system was turned off. Twenty ml of seawater were removed from both injection tubes and discarded to remove any stagnant water. A further 60 ml was removed from the feeding tube and 20 ml from the stimulus tube and retained for flushing. At the start of the 5 min feeding period, we injected 2.5 ml of food (an *Artemia* solution containing ∼250 individuals per ml), followed by 20 ml of seawater (to completely flush the food into the tank), allowing the fish to reach a stable feeding rate before the pre-stimulus observation. At the start of the pre-stimulus observation, an additional 2.5 ml of food was introduced and flushed with 20 ml of seawater. Following the pre-stimulus observation period, we injected 2.5 ml of food, flushed with 20 ml of water, followed by 30 ml of stimulus odour, flushed with 20 ml of seawater. The stimulus odour consisted of the odour from one of the four species used in the ‘cocktail’ or one of the two novel species.

The behaviour of the fish was observed during the pre- and post-observation periods. We quantified three response variables: foraging rate, distance from shelter and time in shelter. Decreased foraging rate and distance from shelter and increased shelter use are well known antipredator responses in a number of prey species, including coral reef fishes [31], [39].The foraging rate included all feeding strikes irrespective of whether they were successful at capturing prey. For distance from shelter, the horizontal and vertical locations of the fish in the tank was recorded every 15 s, using the grid drawn on the side of the tank. The position of the fish in the tank was then converted into a linear distance from shelter using the dimensions of the grid squares (57×42 mm) and Pythagoras's theorem. Time in shelter (in seconds) was defined as total time that the fish spent within one body length of the terracotta pot.

### Statistical analysis

The changes between the pre- and post-stimulus behavioural measures were computed and used as our raw data. The effects of conditioning (alarm cues vs. seawater) and testing odours (the six fish odours) were assessed using a 2-factor MANOVA on all three behavioural responses. Univariate ANOVAs revealed that only one behaviour (foraging) was affected by treatments, so the subsequent analyses were done on the foraging variable only. Because of a significant interaction between the 2 factors, we performed two 2-factor ANOVAs, one testing the effect of conditioning and the cocktail odour only, and one testing the effect of conditioning and the non-cocktail odour, on the responses of the fish. Residual analyses revealed that all data met the assumptions of homogeneity of variance and normality.

## Results

**Figure 1:**
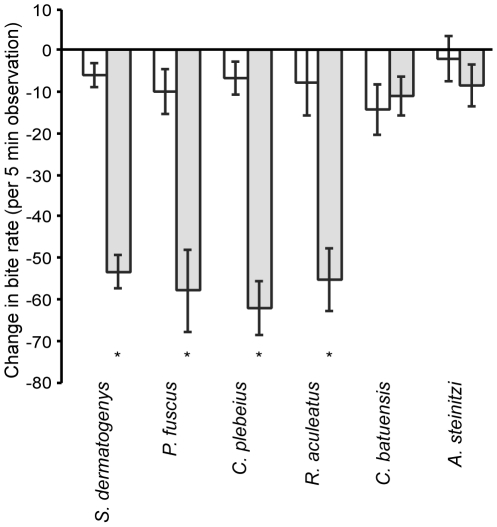
Change in foraging rate for *Pomacentrus moluccensis* in response to different odours. *Pomacentrus moluccensis* were conditioned with the ‘cocktail’ of *S. dermatogenys*, *P. fuscus*, *R. aculeatus* and *C. plebeius* paired with a) alarm cue (shaded bars) or b) saltwater (open bars) and tested for learned recognition of *S. dermatogenys*, *P. fuscus*, *R. aculeatus* and *C. plebeius* odours alone or the control odours of *C. batuensis* and *A. steinitzi*. * indicate significant differences between conditioning treatments within species.

The 2-factor MANOVA revealed a significant interaction between conditioning cues and testing odours on the behavioural response of *P. moluccensis* (conditioning×species, *F*_15, 458.7_ = 3.3, *p*<0.0001). Univariate exploration revealed that foraging was the only behaviour affected by the treatments (Table 1). The 2-factor ANOVAs performed on the ‘cocktail’ odours only revealed a significant effect of conditioning (*F*_1,112_ = 116.0, *p*<0.0001), but no effect of species (*F*_3,112_ = 0.2, *p* = 0.880) and no interaction between the two factors (*F*_3,112_ = 0.2, *p* = 0.910) on the foraging behaviour of *P. moluccensis*, indicating that the fish learned to recognize the four cocktail species as threatening, and responded to all four with the same intensity (fig. 1). Conversely, the 2-factor ANOVA performed on the response to two novel odours revealed no effects of conditioning (*F*_1,56_ = 0.1, *p* = 0.770), no effect of species (*F*_1,56_ = 1.9, *p* = 0.168) and no interaction between the two factors (*F*_1,56_ = 0.8, *p* = 0.368), indicating that the fish did not show an antipredator responses to those 2 odours (fig. 1).

**Table 1:** Univariate results from the 2-factor ANOVA on the effects of Conditioning and Species on behaviour.

Behaviour	Source of Variation	*df*	MS	*F*	*p*
**(a) ** ***Foraging rate***	Species	5	4603.1	8.151	<0.0001
	Conditioning	1	50601.8	89.606	<0.0001
	Species*Conditioning	5	4738.0	8.390	<0.0001
	Error	168	564.7		
**(b) ** ***Time in shelter***	Species	5	7.36	0.504	0.773
	Conditioning	1	9.34	0.640	0.425
	Species*Conditioning	5	6.99	0.479	0.791
	Error	168	14.59		
**(c) ** ***Distance from shelter***	Species	5	10.392	1.069	0.380
	Conditioning	1	9.274	0.954	0.330
	Species*Conditioning	5	5.222	0.537	0.748
	Error	168	9.725		

Comparison of the behaviour of juvenile *Pomacentrus moluccensis* in response to the odours of 6 fish species (‘Species’) after being conditioned with ‘cocktail’ odour paired with either a chemical alarm cue or saltwater (‘Conditioning’).

## Discussion

Our results highlight that juvenile reef fish that are naïve to predators have the ability to rapidly learn multiple unknown predators upon recruitment to the reef. *Pomacentrus moluccensis* recruits conditioned with a ‘cocktail’ of four odours (predators- *S. dermatogenys* and *P. fuscus* and non-predators- *R. aculeatus* and *C. plebeius*) paired with an alarm cue responded with a clear antipredator response when presented the individual odours from the ‘cocktail’, whereas individuals conditioned with the ‘cocktail’ paired with saltwater did not respond. The learning occurred after a single conditioning event. This is the first study to demonstrate rapid learning of multiple predator cues by marine organisms transitioning to a new environment containing multiple novel predators.

The ability to simultaneously learn the identity of multiple predators is an efficient mechanism that allows prey to rapidly garner information regarding predation risk. Acquired recognition of predator odours enhances an individual's survival during encounters with predators whether learnt individually or simultaneously as part of a multi-predator ‘cocktail’ [27]. Such rapid learning is especially important for reef fishes at the time of settlement. The dispersive nature of planktonic larval reef fishes means that juveniles may settle on non-natal reefs, where the diversity and composition of predators may differ from that of their natal reefs [40]. During the first two days post settlement, as individuals learn and adapt to their new environment, mortality due to predation is at its most severe [29]. Faced with such intense predation pressure, an individual's ability to rapidly acquire predator recognition will ultimately determine who survives.

Prey displayed a clear antipredator response to the introduction of a ‘cocktail’ odour following a conditioning event where the ‘cocktail’ odour was paired with the alarm cue. The antipredator response was defined by a substantial decline in the foraging rate of individuals. Reductions in foraging rate in response to predator odours has been demonstrated for the closely related *Pomacentrus amboinensis* [31] and in other species across several taxa in both marine [41], freshwater [42] and terrestrial habitats [43]. Such reductions represent a shift in the balance between foraging and antipredator defence in response to an increase in the perceived risk of predation represented by recognition of an odour associated with a potential predator [12], [44].

*Pomacentrus moluccensis* did not appear to display an antipredator response suggestive of prior recognition to any predator odours used in this experiment. Individuals conditioned with the ‘cocktail’ and saltwater did not respond to individual ‘cocktail’ odours alone. In addition, fish conditioned under either conditioning regime did not show any significant response to the novel odour of *C. batuensis*, a known predator of juvenile reef fish [45]. Recent studies on juvenile pomacentrid recruits suggest that individuals have some level of prior knowledge of predators during settlement, as naïve juveniles avoided predator odours in pairwise Y-maze trails [38] and recruits preferentially settled on habitats where the odour of predators was absent [37]. Prior knowledge of predator odours may be used during site selection by *P. mollucensis* but the odours represent a predation risk that is below their behavioural threshold and do not elicit a measurable anti-predator response until presented in combination with an alarm cue. Further work is needed to identify if pomacentrids have prior knowledge of predator odours at settlement, and if so, how this onboard knowledge is used within the decision-making framework to efficiently balance the costs and benefits of antipredator responses.

The antipredator response of *P. moluccensis* to the ‘cocktail’ odours was consistent across all odours (for their respective conditioning regime) irrespective of whether they originated from a predator or a non-predator. The consistency of the antipredator response to individual ‘cocktail’ odours is unsurprising given the apparent absence of prior knowledge of predator odours and the conditioning regime used during the associative learning of the ‘cocktail’ odours. Both *P. moluccensis* and glowlight tetras were simultaneously conditioned to all odours in combination with exactly the same concentration of alarm cue. During associative learning events, the strength of response to the predator odour is directly related to the concentration of the alarm cue during conditioning [46], [47]. It therefore follows that in the absence of prior knowledge of predators the response to all odours should be consistent for both predators and non-predators.

Simultaneous assessment of the predation risk posed by multiple predators potentially prevents predator specific information from being assessed. Previous studies have highlighted that prey use alarm cues to assess the levels of risk associated with individual predators and are able to respond in a threat sensitive way [48]. European minnows, *Phoxinus phoxinus*, conditioned to recognise predatory pike, *Esox lucius*, or non-predatory tilapia, *Tilapia mariae*, subsequently responded to pike odour with a stronger antipredator response than to tilapia [20]. The difference was suggested to be a result of recognition of predators compared to non-predators or an artefact of evolutionary experience. As shown in this study, simultaneously conditioning prey fish to several odours results in all the odours being assigned the same level of risk. This may lead to a disproportionate behavioural response to the relative level of risk posed by the predators during future encounters. Two outcomes are possible from multi-species conditioning which will results in a net loss in fitness or survival. Firstly, predators of low risk may be labelled as a high risk and under these circumstances the prey fish will respond with an excessive anti-predator response, resulting in time and energy being wasted on unnecessary anti-predator behaviour. Secondly, predators of high risk may be labelled as a low risk predator and the prey fish will respond to its odour with an insufficient response, resulting in the prey being captured and consumed by the predator. Immediately after learning the identity of unknown predators, there is a clear need for the prey to start to fine tune its assessment of the relative risk posed by the predator during subsequent encounters. Ferrari & Chivers [49] demonstrated that minnows would fine-tune their responses to predators after several encounters but would always place more emphasis on the more recent information.

Studies on associative learning have demonstrated that any unknown stimulus can be recognised as a predation risk through associative learning. In natural environments, fishes are constantly exposed to multiple chemical odours. This study highlights the potential for ecologically irrelevant odours to be learned by association when present during a predation event. Responding to irrelevant cues will negatively impact an individual's fitness. Association of odours can be prevented with prior exposure to odours through latent inhibition [26], [50] and learned irrelevance [51] or through experience and constant reassessment of acquired information [49]. However, for reef fish entering a new environment, prior exposure to odours is not possible and some irrelevant odours will be associated with a predation risk. It may pay at first to be overly cautious and learn all odours associated with an alarm cue as a predation risk when entering a new environment and then slowly learn which of those actually do not represent a threat.
